# Tumor staging using 3.0 T multiparametric MRI in prostate cancer: impact on treatment decisions for radical radiotherapy

**DOI:** 10.1186/s40064-015-1596-0

**Published:** 2015-12-18

**Authors:** Felipe Couñago, Elia del Cerro, Ana Aurora Díaz-Gavela, Francisco José Marcos, Manuel Recio, David Sanz-Rosa, Israel Thuissard, Karmele Olaciregui, María Mateo, Laura Cerezo

**Affiliations:** Department of Radiation Oncology, Hospital Universitario Quiron Madrid, Calle Diego de Velazquez, 1, Pozuelo de Alarcón, 28223 Madrid, Spain; Department of Radiology, Hospital Universitario Quiron, Madrid, Spain; Clinical Department, School of Biomedical Sciences, Universidad Europea, Madrid, Spain; School of Doctoral Studies and Research, Universidad Europea, Madrid, Spain; School of Biomedical Sciences, Universidad Europea, Madrid, Spain; Hospital Universitario Quiron, Madrid, Spain; Departament of Radiation Oncology, Hospital Universitario La Princesa, Madrid, Spain

**Keywords:** Prostate cancer, Staging, Radiotherapy, Multiparametric MRI

## Abstract

To assess and validate the incorporation of the multiparametric magnetic resonance imaging (mpMRI) tumour category (mT-category) to the conventional clinical tumour category (cT-category), in order to guide the radiotherapy (RT) treatment decisions in prostate cancer. In addition, to identify the clinical factors associated to the technique reliability. mpMRI was performed in 274 prostate cancer patients in order to refine the treatment decisions according to PSA, Gleason Score (GS) and cT-category. Comparisons between the cT and mT-category were performed, as well as the impact on the RT treatment [target volume, doses and hormonal therapy (HT)] independently if it was finally performed. Changes in HT indication for intermediate risk were also analyzed. mpMRI validation was performed with pathological staging (n = 90 patients finally decided to join surgery). The mpMRI upstaging range was 86–94 % for any PSA value or GS. Following mpMRI, 32.8 % of the patients (90/274) were assigned to a different risk group. Compared to cT-category, mpMRI identified more intermediate-risk (46.4 vs. 59.5 %) and high-risk (19.0 vs. 28.8 %) prostate cancer patients. This resulted in a higher indication (p < 0.05) of seminal vesicle irradiation (63.5 vs. 70.0 %), inclusion of any extracapsular disease (T3–T4) within the target volume (1.8 vs. 18.2 %), higher doses (65.3 vs. 88.3 %) and HT associated to RT (45.6 vs. 62.4 %). Global accuracy for mpMRI was higher compared to DRE/TRUS (8.9 vs. 71.1 %, p < 0.05). mpMRI reliability was independent of PSA or GS. mpMRI tumor staging significantly modified the RT treatment decisions in all prostate cancer risk groups.

## Background

Clinical tumoural category (cT-category) is a determining factor in prostate cancer management. Thus, cT-category, along with prostate-specific antigen level (PSA) and Gleason score (GS), contribute to establishing tumour recurrence risk groups, and subsequently to making radiotherapy treatment (RT) decisions [such as target volume, total dose, and addition of hormonal therapy (HT)]. In determining the cT-category in prostate cancer, digital rectal examination (DRE) and transrectal ultrasound (TRUS) remain the gold-standard methods recommended in almost all guidelines and protocols (Mohler et al. 2014; Heidenreich et al. 2014). However, both tests have very low reliability and high interobserver variability (Philip et al. 2005; Smith and Catalona 1995). This could result in erroneous estimation of the cT-category, potentially resulting in inappropriate treatment, particularly in relation to RT.

In recent years, multiparametric magnetic resonance imaging (mpMRI) has been the most reliable technique for local tumour staging in prostate cancer patients, compared with conventional tests such as DRE, TRUS and morphological MRI, as well as predictive tools such as the Partin tables and the Kattan nomogram (Hricak et al. 1987; Mullerad et al. 2005; Augustin et al. 2009; Wang et al. 2007). However, few studies have focused on the impact of mpMRI staging in relation to RT target volumes, total dose administered, or the use of HT (Couñago et al. 2014; Panje et al. 2015).

Although heterogeneity is common among institutional protocols (Horsley et al. 2015), at our institution, the recommendation for low-risk patients is to treat the prostate with RT (76–78 Gy) without associated HT; by contrast, for organ-confined (T1–T2) intermediate-risk and high-risk patients, the irradiation of both prostate and seminal vesicles (78–80 Gy) is indicated (Mohler et al. 2014; Heidenreich et al. 2014). In high-risk patients with locally advanced disease (T3–T4), it is also necessary to extend the target volume beyond the prostatic capsule to encompass any type of extracapsular extension (ECE) (Boehmer et al. 2006; Hayden et al. 2010). Long-term HT treatment (≥24 m) is recommended for high-risk patients. Short-term treatment (4–6 m), while controversial, is recommended for intermediate-risk patients with unfavourable prognostic factors (Zapatero et al. 2015; D’Amico 2015). Either prophylactic lymph node irradiation or brachytherapy boost following RT are optional in intermediate-risk and high-risk patients (Mohler et al. 2014; Heidenreich et al. 2014).

With this in mind, the principal aim of our study was to analyse and validate the consequences of mpMRI staging for RT treatment decisions (target volume, doses and HT) in prostate cancer, as well as to determine the clinical factors associated with mpMRI reliability.

## Methods

### Study design

This is a retrospective study conducted in our Oncology Department with the approval of Quiron Hospital’s Ethics Committee. Between January 2009 and April 2015, 274 patients with histological diagnoses of primary prostate cancer, mpMRI-categorized and considered for definitive treatment with RT ± HT or radical prostatectomy (RP), were included in our study. Patients without mpMRI staging prior to any treatment (n = 175) or with non-assessed mpMRI due to bleeding after prostatic biopsy (n = 6) were excluded. Ten patients without cT-category were also excluded. All patients were assessed by Urology and Radiation Oncology Services before the definitive treatment decision. The completion of RT treatment was not essential for inclusion in the study, as decisions regarding target volume, doses, and HT depend exclusively on tumour category, PSA value and GS.

Before treatment was started, patients’ baseline characteristics were evaluated, including age at diagnosis, DRE, PSA value at diagnosis, and a TRUS-guided biopsy from which the patients’ GS were obtained. Other imaging techniques (CT and bone scan) were optional, according to international guidelines (Mohler et al. 2014; Heidenreich et al. 2014) and the physician’s judgment, basically in intermediate and high risk patients. cT-category before mpMRI was assigned on the basis of DRE and TRUS. Tumour category was assessed according to the American Joint Committee on Cancer (AJCC) system, 7th edition (AJCC 2010). Based on PSA values, GS and cT-category, relapse risk groups were established following the National Comprehensive Cancer Network guidelines version 2.2014: low-risk, ≤T2a, GS ≤ 6 and pretreatment PSA <10 ng/mL; intermediate-risk, T2b–T2c or GS = 7 or PSA 10–20 ng/mL; high-risk, T3–T4 or GS >7 or PSA >20 ng/mL; metastatic, N+ or M+ (Mohler et al. 2014).

Based on the prostate cancer clinical protocol in force in our institution’s radiotherapy unit, initial indication (target volume, doses and HT) was established according to cT-category; ultimate indications were based on MRI tumour category (mT-category). Up- or downstaging, changes in risk group, and alterations in RT treatment due to tumour category variations were analysed. In addition, for patients whose final treatment consisted of RP (n = 90), the mT-category was compared to the pathologic tumour category (pT-category), which allowed us to validate mpMRI results.

### mpMRI technique

mpMRI was performed on an eight-channel torso-array antenna 3 Tesla (General Electric, USA) in previously prepared patients. The study protocol was extensively described in a previous study (Couñago et al. 2014). In brief, morphological imaging included T1- and T2-weighted sequences and functional studies included diffusion-weighted imaging (DWI) and dynamic contrast-enhanced (DCE) imaging. The apparent diffusion coefficient (ADC) map was calculated while using DWI. DCE imaging was performed applying contrast with gadolinium. In the DCE study, time-intensity curves were calculated. The mpMRI was performed after conventional clinical staging, and always interpreted by one uroradiologist experienced in pelvic MRI.

### Risk-adapted RT

Patients underwent treatment in a multienergetic lineal accelerator with intensity modulated radiotherapy (IMRT). In low-risk patients, the dose was 76–78 Gy, increasing to 78–80 Gy in intermediate-risk and high-risk patients. The clinical target volume (CTV) included only the prostate in low-risk patients, while the prostate and seminal vesicles were included in intermediate-risk and high-risk patients. CTV was extended to include ECE in T3–T4 patients, following the recommendations of clinical guidelines (Boehmer et al. 2006; Hayden et al. 2010). The pelvis was not included, except in cases with clinical pelvic node involvement. High-risk patients received long-term HT (2–3 years) and intermediate-risk patients with unfavourable factors received short-term HT (4–6 months). Until 2014, the unfavourable factors according to the initial criteria were a GS of 7 (4 + 3), or three unfavourable intermediate risk factors (T2b + PSA 10–20 ng/mL + GS 3 + 4), or T2c by DRE/TRUS; more recently, unfavourable risk factors have been established according to memorial sloan kettering cancer center (MSKCC) criteria: GS 4 + 3, or at least two intermediate-risk factors, or at least one intermediate-risk factor and a positive prostate biopsies (ppb) percentage greater than 50 % (D’Amico 2015; Zumsteg et al. 2013).

### Statistical analysis

The data analysis was performed with IBM SPSS statistics version 21.0 (IBM Corp; USA). Descriptive statistics are reported as median (IQR, interquartile range) or mean (SD, Standard Deviation) for continuous variables, and as absolute or relative frequencies for categorical variables. Patient basal characteristics and the differences in treatment impact on DRE/TRUS vs. mpMRI, as well as the reliability of the diagnostic tests, were compared using the Chi square test and Fisher’s exact test, both for categorical variables. The U Mann–Whitney test was used for the non-parametric quantitative variables. To analyse the relationship between the reliability of mpMRI and the clinical variables, a univariate and multivariate logistic regression analysis was performed.

## Results

**Fig. 1:**
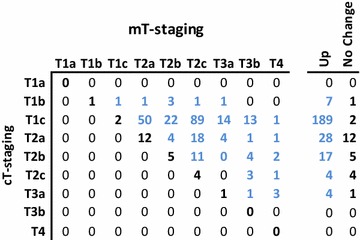
Comparison between cT and mT-staging. The *panel* shows in *bold* *black*, those patients whose clinical stage was similar using both strategies

The baseline patient characteristics of the entire cohort (n = 274) and the surgical patient cohort (n = 90) are summarised in Table 1. Upstaging occurred in 90.9 % of the patients (249/274 patients; see Fig. 1). The upstaging range was 86.1–94.2 % for any PSA or GS value. Downstaging was not observed. Following mpMRI, 32.8 % of the patients (90/274) were assigned to a different risk group. Finally, decisions concerning RT were changed in 43.8 % (initial criteria) or 52.5 % (MSKCC criteria) of the patients, depending on the criteria applied to indicate HT in intermediate-risk patients.

**Table 1:** Patients characteristics

Characteristics	Total cohort (%)	Radical prostatectomy (%)
Patients	274 (100)	90 (100)
Age (years)	66.8 ± 8.1	60.9 ± 6.8
PSA (ng/mL)		
<10	179 (65.3)	68 (75.6)
10–20	66 (24.1)	18 (20.0)
>20	29 (10.6)	4 (4.4)
Gleason score		
≤6	137 (50.0)	60 (66.7)
7	108 (39.4)	24 (26.7)
≥8	29 (10,6)	6 (6.6)
cT-category before mpMRI		
≤T2a	239 (87.2)	84 (93.3)
T2b–T2c	30 (11.0)	6 (6.7)
T3a	5 (1.8)	0
T3b	0	0
T4	0	0
Risk groups		
Low	95 (34.7)	43 (47.8)
Intermediate	127 (46.4)	37 (41.1)
High	52 (19.0)	10 (11.1)
Positive prostate biopsies		
<50 %	158 (57.7)	49 (54.4)
≥50 %	65 (23.7)	16 (17.8)
(Non classified)	51 (18.6)	25 (27.8)

*mpMRI* multiparametric magnetic resonance imaging*, PSA* prostate-specific antigen

Upstaging from cT1–T2a to mT2b-T2c occurred in 137 patients of a total of 239 patients (57.3 %). 58 out of 239 patients (24.2 %) upward shift from low-risk to intermediate-risk resulting in a modification of target volume due to the inclusion of seminal vesicles, and the increase in dosage from 76–78 to 78–80 Gy. In 7 of a total of 11 of the intermediate-risk patients (cT1–cT2a and GS 3 + 4 and PSA 10–20 ng/mL), short-term HT was also indicated, as 3 unfavorable risk factors were present. However, if the most recent MSKCC criteria had been applied, 31 out of 41 intermediate-risk patients [cT1–cT2a and (GS 3 + 4 or PSA 10–20 ng/mL or ppb >50 %)], would have received short-term HT.

Among the 269 cT1–T2 patients, 45 (16.7 %) progressed to mT3–T4, based on the detection of any type of ECE in mpMRI. This entailed an alteration of target volume due to the inclusion of seminal vesicles, as well as ECE, an increase in dosage, and inclusion of long-term HT for five low-risk patients. Of these 45 patients, 22 were allocated to the intermediate-risk group, resulting in the modification of the target volume due to the inclusion of ECE and the addition of long-term HT. Lastly, the inclusion of ECE in the target volume was the only alteration in 18 high-risk patients.

Of a total of five cT3a patients, one progressed to mT3b, but this case did not imply modifications of final RT treatment decisions, as in high-risk patients seminal vesicles are always irradiated at full doses according to our institution’s protocol. Of the remaining four cT3a patients, three progressed to mT4 and consequently were treated with an increase in target volume to enable infiltration of the rectum or the bladder. Lastly, out of 127 intermediate-risk and 52 high-risk patients, stage IV was diagnosed in two and four patients respectively (3.4 %). In these patients, treatment consisted of either HT exclusively, or pelvic RT and long-term HT.

In general, the mpMRI staging led to the identification of more intermediate-risk and high-risk patients compared to conventional clinical staging. As a result, a higher occurrence of irradiation of seminal vesicles, inclusion of any ECE (T3–T4), and more frequent prescription of combined RT/HT, as well as higher doses of RT (p < 0.05), were observed (Table 2).

**Table 2:** Impact of mpMRI tumor staging on RT treatment prescription (target volume, doses and hormonal therapy)

Characteristics	DRE/TRUS n (%)	mpMRI n (%)	p value
*T-category*			
T1–T2a	239 (87.2)	67 (24.5)	*<0.001*
T2b–T2c	30 (11.0)	157 (57.3)	
T3–T4	5 (1.8)	50 (18.2)	
*Risk group*			
Low-risk (LR)	95 (34.7)	32 (11.7)	*<0.001*
Intermediate-risk (IR)	127 (46.4)	163 (59.5)	
High-risk (HR)	52 (19.0)	79 (28.8)	
*Target volume*			
VVSS prophylactic	174 (63.5)	192 (70.1)	*<0.001*
T3a + T3b + T4	5 (1.8)	50 (18.2)	
*Doses*			
Low	95 (34.7)	32 (11.7)	*<0.001*
High	179 (65.3)	242 (88.3)	
*Hormonal therapy*			
HR and IR (Initial criteria)^a^			
No	182 (66.4)	158 (57.7)	*0.035*
Yes	92 (33.6)	116 (42.3)	
HR and IR (MSKCC criteria)^b^			
No	149 (54.4)	103 (37.6)	*<0.001*
Yes	125 (45.6)	171 (62.4)	
*Hormonal therapy in IR patients*			
Initial criteria			
No	220 (80.3)	221 (80.7)	0.914
Yes	54 (19.7)	53 (19.3)	
MSKCC criteria			*0.002*
No	180 (65.7)	144 (52.6)	
Yes	94 (34.3)	130 (47.4)	
*Hormonal therapy in HR patients*			
No	222 (81.0)	195 (71.2)	*0.007*
Yes	52 (19.0)	79 (28.8)	

*VVSS* seminal vesicles*, DRE/TRUS* digital rectal exam/transrectal ultrasound

^a^ *Initial criteria:* GS of 7 (4 + 3), or three unfavourable IR factors (T2b + PSA 10-20 ng/mL + GS 3 + 4), or T2c by DRE/TRUS

^b^ *MSKCC criteria:* GS 4 + 3, or at least two IR factors, or at least one IR factor and a positive prostate biopsy (ppb) percentage greater than 50 %

In order to validate the mpMRI results, 90 patients treated with RP were analysed. The mT-category and the pT-category were compared (Table 3). Global accuracy of cT-staging with DRE/TRUS was 8.9 % (8/90), while it was 71.1 % (64/90) for mpMRI. Among the 51 patients upstaged from cT1–T2a to mT2b–T2c, 38 patients (74.5 %) showed MRI findings in agreement with the pathological staging. For the 12 patients upstaged from cT1–T2 to mT3, this agreement occurred in seven patients (58.3 %). This entailed a 91.1 % accuracy, 70.0 % (95 % CI 41.6–98.4) sensitivity, 93.8 % (95 % CI 88.5–99.1) specificity, 58.3 % positive predictive value (95 % CI 30.4–86.2) and a 96.2 % negative predictive value (95 % CI 91.9–100) in the MRI staging of ECE (T3) patients.

**Table 3:** mT-staging validation for the indication of RT treatment

	DRE/TRUS n (%)	mpMRI n (%)	*p* value	Prostatectomy piece n (%)
*T-category*				
T1–T2a	84 (93.7)	24 (26.7)	*<* *0.001*	23 (25.6)
T2b–T2c	6 (6.7)	54 (60.0)		57 (63.3)
T3–T4	0	12 (13.3)		10 (11.1)
*Risk group*				
Low-risk	43 (47.8)	13 (14.4)	*<* *0.001*	10 (11.1)
Intermediate-risk	37 (41.1)	59 (65.6)		65 (72.2)
High-risk	10 (11.1)	18 (20.0)		15 (16.7)
*Global reliability for T-category*	8 (8.8)	64 (71.1)	*<* *0.001*	
*Risk group change due to pathological analysis*	37 (41.1)	15 (16.7)	*<* *0.001*	
*Changes related to RT parameters (HT, Doses, CTV)*				
Initial criteria	45 (50.0)	16 (17.8)	*<* *0.001*	
MSKCC criteria	59 (65.6)	18 (20.0)		

For patients whose final treatment consisted of RP (n = 90), the cT-and mT-category were compared to the pathologic tumour stage (pT-category), which allowed us to validate DRE/TRUS and mpMRI results. Furthermore, the analysis and comparisons related to RT treatment decisions were done for DRE/TRUS vs. mpMRI

*RT* radiotherapy, *HT* hormonal therapy, *CTV* clinical target volume

Having the pT-staging as the gold standard, there was less disagreement in the RT treatment decisions (CTV, HT indication, RT prescription dose) with mT-staging compared to cT-staging (Table 3).

### Clinical factors associated to mpMRI reliability

Possible clinical variables associated with an increase in mpMRI reliability were analysed in relation to tumour staging through univariate and multivariate logistic regression analysis. None of the clinical factors analysed (PSA value, GS,  % ppb and age) showed a significant association with mpMRI reliability (Table 4).

**Table 4:** Multivariate analysis of clinical factors associated to mpMRI reliability

	OR	95 % CI	p-value
PSA <10	Reference		
PSA 10–20	0.43	(0.03–5.05)	0.50
PSA >20	0.61	(0.04–8.21)	0.70
Gleason ≤6^a^	Reference		
Gleason ≥7	1.39	(0.41–4.65)	0.58
ppb <50 %	Reference		
ppb ≥50 %	1.07	(0.31–3.69)	0.90
Age	0.92	(0.84–1.00)	0.06

*ppb* positive prostate biopsies

^a^No significant differences were found when considering Gleason ≤6 and Gleason = 7 (3 + 4) compared to the rest of Gleason score. Similar lack of significant differences were observed when Gleason = 6 was compared to Gleason = 7 or Gleason = 8–10

## Discussion

We believe that this is the largest cohort of patients in which the impact of mpMRI staging on RT treatment has been analysed. In brief, 90.9 % of patients showed tumour upstaging as a result of mpMRI. Nevertheless, when considering all the risk factors together, such as PSA levels, GS, and tumour category, a change in the risk groups occurred in only 32.8 % of patients. In our patient series, upstaging derived from mpMRI modified RT treatment for three main reasons: alteration of a patient’s risk group; extension of target volume due to any type of ECE (T3–T4 patients) in high-risk patients; or treatment with short-term HT due to accumulation of unfavourable factors in intermediate-risk patients. While there are proven benefits in overall survival (OS) among high-risk patients of long-term HT associated with high-dose RT (Zapatero et al. 2015), these benefits are not clear in all intermediate-risk patients (D’Amico 2015). With the available scientific evidence on the matter, many authors recommend associating HT with high-dose RT only in intermediate-risk patients with unfavourable factors (D’Amico 2015), and this is the protocol we have applied at our institution.

Very few studies have evaluated the impact of MRI staging on final decisions in RT treatment (Table 5). We obtained a global alteration of RT treatment in 43.8 or 52.5 % of patients (depending on HT criteria for intermediate-risk patients). Other studies have shown a change in RT treatment of between 8 and 34 %. Such variability can be due to several causes: some studies included patients who underwent MRI after starting HT treatment, leading to a reduction of tumour size and consequently a false downstaging (Panje et al. 2015; Chang et al. 2014); on the other hand, most of the studies in this context were performed using 1.5T MRI without endorectal coil (Panje et al. 2015; Yamaguchi et al. 2015; Chang et al. 2014; Jackson et al. 2005) whereas our investigation was conducted using 3T MRI, with higher diagnostic reliability (Park et al. 2007). Morphological MRI has been used in most of the reported series without functional associated studies (Horsley et al. 2015; Yamaguchi et al. 2015; Chang et al. 2014; Jackson et al. 2005). Our study implemented the guide-recommended (Barentsz et al. 2012) morphological T2 sequences along with two functional studies (DWI and DCE), increasing the diagnosis rate, as previously reported (Wu et al. 2012; Verma et al. 2012). In particular, we observed a large number of patients initially categorized as cT1–T2a (239 patients), which might have contributed to such a marked degree of upstaging. On the other hand, in many of the aforementioned studies, HT was administered to all intermediate-risk patients (Panje et al. 2015; Horsley et al. 2015; Yamaguchi et al. 2015; Chang et al. 2014; Jackson et al. 2005), independent of unfavourable risk factors. In this respect, if we had considered administering HT to all intermediate-risk patients, the impact of RT treatment would have decreased to 41.2 %. On the other hand, in the literature, conventional clinical staging was performed not only using DRE/TRUS, but also with pelvic CT (Panje et al. 2015), which could have influenced the final results. Lastly, it must be remarked that patients who progressed to the metastatic category due to pelvic node involvement and/or bone metastases were taken into account. In some studies, these patients were excluded from the final analysis (Horsley et al. 2015).

**Table 5:** Clinical studies evaluating the impact of the staging using MRI in PCa patients treated with RT

Study	Type of MRI	n	Field strength	Coil	Tumor stage shift ( %)	Risk group changes ( %)	Change in RT (CTV, doses, HT) ( %)	Technique validation
Panje et al. (2015)	Multiparametric	122	1.5 T & 3 T	PAB	55.7	28.7	30	No
Horsley et al. (2015)	Morphological	509	1.5 T	PAB	20	9	18	No
Yamaguchi et al. (2015)	Morphological	157	1.5 T	PAB	25	9	8^a^	No
Couñago et al. (2014)	Multiparametric	103	3 T	PAB	94.1	33.9	33.9	Yes
Chang et al. (2014)	Morphological	115	1.5 T	PAB	68.6	7	20^a^	No
Jackson et al. (2005)	Morphological	199	1.5 T	PAB	55	NR	32.6^b^	No
Present study	Multiparametric	274	3 T	PAB	90.4	32.8	43.8 or 52.5^c^	Yes

Not RT change reported

*PAB* Phased-array-bodycoil, *NR* not reported, *CTV* clinical target volume, *HT* hormonal therapy

^a^Exclusive assessment of the CTV change

^b^Data from T1–T2 to T3–T4 upstaging

^c^Values according to the HT criteria in intermediate-risk patients

The greatest impact of mpMRI on RT treatment prescription occurs in low-risk or intermediate-risk cT1–T2 patients who progress to mT3–T4, due to the fact that in addition to expanding the target volume to include ECE, we must associate long-term HT with the increased toxicity this entails. Our study shows that 16.7 % of patients were upstaged to T3–T4. This result is similar to that found by Horsley et al.(2015), who investigated the impact on RT treatment prescription of staging using morphological 1.5 T MRI with body-coil. In their study of 509 patients, Horsley et al. found that 20 % of patients initially categorized as T1–T2 were upstaged to T3–T4 based on MRI findings. On the other hand, Jackson et al. (2005) obtained a 35.2 % change in treatment decision due to MRI findings in T3–T4 patients. This value can be seen as unrealistic, given the fact that in our surgical patient cohort, mpMRI testing showed a low predictive positive value (58.3 %) for T3 staging; this potentially constitutes an over-diagnosis, and consequent over-treatment and increased toxicity in patients. These data are consistent with a recent meta-analysis that warns of the low sensitivity of MRI in ECE detection (de Rooij et al. 2015). However, a limited number of patients would be under-treated due to the high negative predictive value (96.2 %) for any ECE detection. In contrast, we obtained 71.0 % for overall accuracy with mpMRI staging, while the value for DRE/TRUS was only 8.8 %. Globally, mT staging allowed us to prescribe RT treatments with higher reliability than did cT-staging.

In our series, the global change within the patient risk group was analysed considering the tumour category, as well as the PSA level and the GS. Ours results are comparable to a recent paper published by Panje et al. (32.8 vs 28.7 %) (Panje et al. 2015). These data suggest that a minimum of approximately 30 % of prostate cancer patients staged with mpMRI could face an alteration of the final RT treatment decision. The ultimate impact on treatment prescription will vary according to the different care protocols in prostate cancer management, such as the criteria to include the seminal vesicles or pelvis within the target volume, administered treatment doses, HT criteria in intermediate-risk patients, and the use of combined brachytherapy with RT. Unfortunately, all these issues are very variable among different institutions (Horsley et al. 2015).

When analysing which patients would benefit most from undergoing MRI for staging purposes, our study, along with those of Panje and Horsley (Panje et al. 2015; Horsley et al. 2015), found significant upstaging in all risk groups—in our case, 86–94 % for any PSA or GS value. In addition, we observed that 66.3 % of low-risk patients progressed to intermediate-risk or high-risk; 18.1 % of intermediate-risk patients progressed to high-risk or metastatic; and 7.7 % of high-risk patients were upstaged to metastatic. Also, of the 45 patients upstaged to T3–T4 because of mT-staging, 11.1, 48.9 and 40.0 % were initially low-risk, intermediate-risk, and high-risk, respectively. Lastly, though there are published studies that suggest that tumour detection and location are worse the lower the GS value and tumour size are (Bratan et al. 2013), our univariant and multivariant logistic regression analysis showed no clinical factors associated with lower technique reliability in tumour staging. Consequently, in our clinical environment, all patients with localised prostate cancer who are being considered for RT will benefit from mpMRI staging.

This study presents the following limitations: first, it is a retrospective study with all the inherent limitations associated with this design; second, our follow-up time was not long enough to validate the prognostic significance of upstaging through mpMRI, although this was not the main aim of the study; third, as mpMRI is more accurate than DRE/TRUS and led to a change in T-category or management, this did not lead to increased survival in patients. This hypothesis should be confirmed in a phase three clinical trial; fourth, the radiologist was not blinded, and consequently knew the patients’ initial clinical category before the MRI took place. This bias was minimised when quantitative parameters such as ADC or semi-quantitative ones such as time-intensity curves were analysed. In addition, this is common clinical practice and is assumed in most published studies on this matter (Panje et al. 2015; Horsley et al. 2015; Yamaguchi et al. 2015; Chang et al. 2014; Jackson et al. 2005). Lastly, DRE/TRUS reliability was found to be very low in our study (8.8 %), and this could have influenced the elevated upstaging values observed with mpMRI. Having said this, the literature on this subject describes very low reliability for DRE/TRUS in tumour staging (<50 %), and high interobserver variability (Philip et al. 2005; Smith and Catalona 1995). However, the impact of upstaging on risk-group modification (according to all factors—T-staging, PSA and GS) was similar to other study using mpMRI (Panje et al. 2015).

In conclusion, mpMRI tumour staging significantly modified the RT treatment decisions in all prostate cancer risk groups. Approximately 30 % of patients may experience a change in their initial risk group based on mpMRI findings. The magnitude of the impact on final RT treatment decisions will depend on the institution’s clinical protocol for prostate cancer management.
